# Case Report: Successful immune checkpoint inhibitor-based rechallenge in a patient with advanced renal clear cell cancer

**DOI:** 10.3389/fimmu.2023.1270828

**Published:** 2023-10-17

**Authors:** Shengxiu He, Liang Wang, Yan Sun, Huakun Du, Xiaomin Yu

**Affiliations:** ^1^ Division of Medical Oncology, Dalian Municipal Central Hospital, Dalian, China; ^2^ Medical Affairs Department, Guangzhou Gloria Biosciences Co. Ltd., Beijing, China

**Keywords:** zimberelimab, immune checkpoint inhibitor, rechallenge, advanced renal clear cell cancer, immune-related adverse events

## Abstract

With the rapidly evolving of immune checkpoint inhibitors (ICIs), it has shown remarkable clinical benefits in treating various cancers. However, immune-related adverse events (irAEs) remain a significant challenge in the management of patients undergoing immunotherapy. There are limited data about immunotherapy re-challenge in patients with renal clear cell cancer who had irAE in the initial ICI therapy. In this study, we reported the case of a patient with advanced renal clear cell cancer who developed serious irAEs but also achieved a partial remission of tumor after ICI combined with pazopanib in the first-line treatment. After intravenous methylprednisolone therapy for two weeks, the patient fully recovered from treatment-related toxicities. After a multidisciplinary treatment (MDT) discussion and a communication with the patient, the decision was made to undergo a new fully humanized programmed death 1 (PD-1) agent, zimberelimab, combined with pazopanib for immune restart therapy. After two cycles of treatment, the patient demonstrated a partial response (PR), and the disease remained in continuous remission without any irAE at our last follow-up after 14 months’ treatment. Re-challenging with immunotherapy after irAEs is an emerging strategy that offers the potential for additional clinical benefits to previously responding patients. However, careful patient selection and monitoring are essential to maximize the safety and efficacy of this approach.

## Introduction

With the rapidly evolving of immune checkpoint inhibitors (ICIs), it has shown remarkable clinical benefits in treating various cancers (1). However, immune-related adverse events (irAEs) remain a significant challenge in the management of patients undergoing immunotherapy. IrAEs can occur in nearly every organ system in the body, limiting the tolerability and duration of the therapy (2). Despite this, re-challenging with immunotherapy has emerged as a promising approach in selected patients who have previously experienced irAEs (3).

ICIs Re-challenging therapy involves resuming treatment after an irAE-related discontinuation or holding treatment for a defined period to let the irAE resolve (4). The rationale behind this strategy stems from the observation that irAEs often accompany a response to immunotherapy, indicating that immune activation is occurring (5).

Most irAEs were typically reversible after discontinuation of treatment and treatment with steroids (6). Current cancer treatment guidelines recommend permanent discontinuation of ICI therapy only for the most severe adverse reactions (grade 4). Thus, it appears that immune therapy-related adverse reactions may recur upon resuming treatment following a temporary drug discontinuation. However, limited data could be available regarding the safety of reinitiating treatment in cases of severe immune-related adverse events (7).

Herein, we report a case of successful immune checkpoint inhibitor-based rechallenge in metastatic renal cell cancer after it had been discontinued due to irAEs.

## Case description

**Figure 1:**
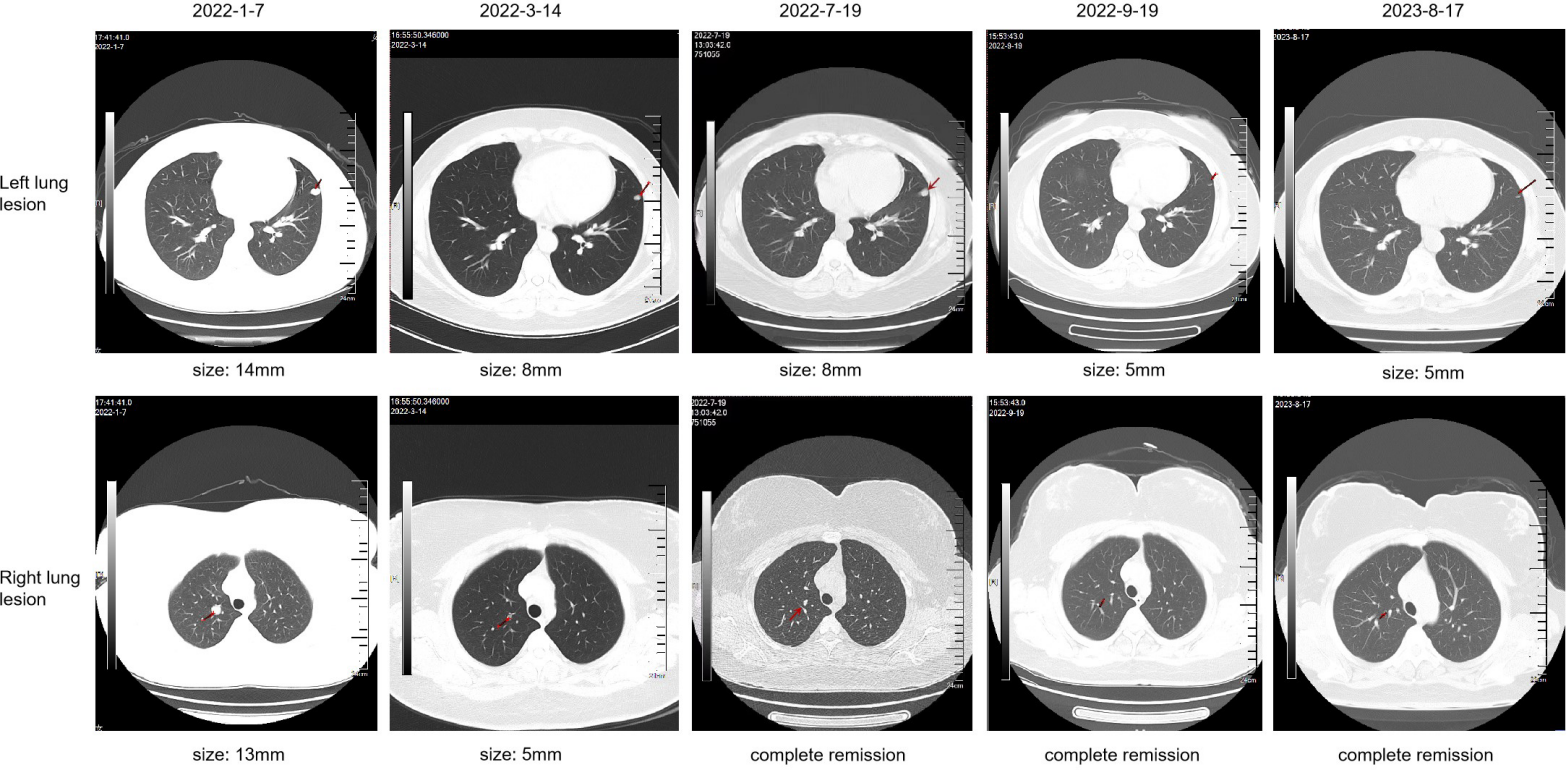
Computed tomography (CT) imaging of lung lesions at critical time point during the treatment course.

A 34-year-old female patient presented with backache and hematuria and subsequently underwent computed tomography (CT) imaging at the Third Hospital of Wafangdian, revealing a right kidney lesion. The measured size of the lesion was 7.8*8.5*10.7cm with no lymphatic metastasis. Additionally, a small nodule of 8mm was detected in the left lower lung. On April 13^th^, 2021, the patient underwent laparoscopic radical nephrectomy. The postoperative pathological diagnosis confirmed clear-cell renal carcinoma with an International Society of Urological Pathology (ISUP) grade II. The resected tumor sample was completely contained within the renal parenchyma, measuring 8*8*4cm in size, and the ureter was uninvolved. The patient had an uneventful postoperative recovery without any adjuvant therapy. However, on January 7^th^, 2022, the patient was transferred to our center and CT scan of the lungs showed an enlargement of the pre-existing left lower lung nodule compared to April 11^th^, 2021, as well as the appearance of a new right pulmonary nodule. The maximum diameters of these two nodules were 13mm and 14mm for the right and the left lung, respectively (**Figure 1**). Based on the medical history and imaging findings, the possibility of metastatic disease was considered. Consequently, the patient received a single dose of sintilimab 200mg and pazopanib 800mg daily orally on January 8^th^, 2022.

On the fifth day of drug administration, the patient presented with fever of grade IV, which peaked at a maximum body temperature of 40.6°C. Febrifuge was given immediately, which was effective at first. However, after four days, neither antibiotic nor febrifuge could control the temperature, and the patient experienced shock caused by persistent high fever. On January 22^nd^, considering the fever could be immune-related, the patient was given 80mg intravenous methylprednisolone therapy, and the temperature decreased temporarily. The body temperature rose again in the afternoon and other symptoms emerged in succession in the following 2 days, including:

1) hepatic impairment of grade III;
2) hypoalbuminemia of grade II;
3) thrombocytopenia of grade III, leading to coagulopathy and subcutaneous hemorrhage;
4) ocular toxicity of grade III blurred vision, diagnosed with keratitis after ophthalmologic consultation.

These clinical features indicated immune-related toxicity, and the antitumor medication was discontinued immediately. On January 24^th^, the patient started receiving 80mg intravenous methylprednisolone therapy twice a day for 10 days, which led to a gradual resolution of all the symptoms. Subsequently, the patient developed concomitant hypertension, with the highest blood pressure reaching 240/180mmHg, requiring antihypertensive medication and a reduction in the dose of pazopanib from 400mg to 200mg to effectively manage the patient’s condition.

**Figure 2:**
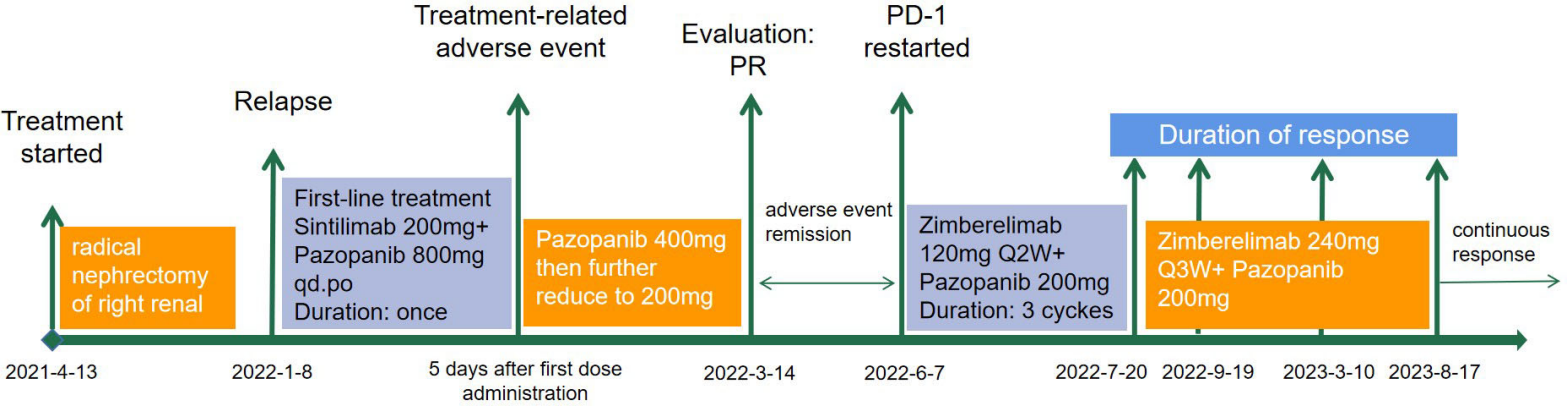
The treatment history of a patient with advanced clear-cell renal carcinoma treated with PD-1 rechallenge.

The patient achieved partial response (PR) on March 14^th^ (**Figure 1**), with significant shrinkage of both lesions: the left lung lesion was measuring 8mm and the right lung lesion 5mm. The patient was experiencing difficulties in controlling the high blood pressure and was concerned about the potential impact of reducing the pazopanib dose to 200mg. After a multidisciplinary treatment (MDT) discussion and a communication with the patient and their family, the decision was made to undergo immune restart therapy. Treatment was initiated on June 7^th^, with zimberelimab at a dosage of 120mg every 14 days and pazopanib at a dosage of 200mg. The patient’s blood pressure was generally well-controlled. After three doses, a clinical evaluation was conducted on July 19^th^, which revealed a complete remission of the right lung lesion, while the left lung lesion remained stable. The patient demonstrated good tolerance to the treatment. The dosage was then increased to 240mg (2 doses) every 3 weeks, and a subsequent efficacy evaluation on September 19th, after two cycles of treatment, indicated a response of left lung lesion to 5mm (**Figure 1**). The disease remained in continuous response at our last follow-up, August 17^th^, 2023 (**Figure 1**). Surprisingly, there were no immune-related adverse reactions during the treatment. Entire treatment history of the patient was showed in **Figure 2**.

## Discussion

Due to the lack of reliable predictive and prognostic factors for the recurrence of immune-related adverse events (irAEs), it posed a challenge in cases after ICI re-administration. Charles Dolladille et al. suggested, in such cases who had severe irAEs, the restart of ICI therapy decisions should take into account the type, grade, and timing of irAEs, response to immunosuppression therapy, life expectancy, performance status, comorbid conditions, patient preferences, and alternative cancer therapy options, among other determinants (7). It is essential to conduct a thorough evaluation of these factors by experts before considering the resumption of ICI therapy.

Renal cell carcinoma (RCC) is sensitive to immunotherapy and antiangiogenic tyrosine kinase inhibitors. Combination of PD-1 antibody and vascular endothelial growth factor (VEGF) inhibitor has become the standard therapy for metastasis RCC, such as axitinib plus avelumab or pembrolizumab, cabozantinib plus nivolumab and lenvatinib plus pembrolizumab (8). For our patient, treatment options are limited. Historically, cytotoxic chemotherapy had little to no activity in patients with RCC. And since this patient was diagnosed with hypertension of grade 3, usage of VEGF antibodies needs to be more cautious as VEGF receptor inhibitors have been reported to induce hypertension (9). Considering that the patient achieved PR in previous ICI therapy, the occurrence of serious adverse events (SAEs) in the first therapy could be attributed to the efficacy of treatment, as previous study demonstrated that patients who developed irAEs experienced both an overall survival (OS) benefit and a progression-free survival (PFS) benefit from ICI therapy compared to those who did not (5). Overall, there’s a great chance that rechallenge with ICI-based therapy could improve survival. Therefore, we take re-challenge ICI as our primary treatment option after MDT and after the communication with the patient.

Previous studies demonstrated that ICI re-challenge was feasible with manageable results. A database analysis study suggested that 61.1% of the patients who discontinued ICI treatment for grade ≥2 irAEs experienced no recurrent grade ≥2 irAEs after ICI rechallenge (10). And in other 2 retrospective studies of metastatic melanoma and non-small cell lung cancer, recurrence rate of initial irAEs were 18% (11) and 26% (12), respectively. Retrospective studies and case reports of other cancer types have also demonstrated that rechallenge with immune checkpoint inhibitors (ICIs) results in a survival benefit for patients, with the consequent adverse events typically being well-tolerated (13–15).

In our case, the choice of ICI for re-challenge is zimberelimab, a new fully humanized PD-1 antibody, which has been developed using the OmniRat platform (16). This approach offers several benefits over traditional antibody development methods such as hybridomas or phage display. For instance, it can overcome the limitations of traditional methods, including the inability to generate fully human antibodies, difficulty in obtaining high-affinity antibodies, and low-yield production. Additionally, using the OmniRat platform has the potential to reduce the risk of immunogenicity and other adverse reactions that can occur when using non-humanized antibodies (17). Therefore, zimberelimab has the potential for improved efficacy and decreased risk of adverse reactions when compared to partially humanized antibodies.

However, ICIs re-challenging therapy is not without its potential risks, and close monitoring by the healthcare team is necessary throughout the re-challenge period. Especially in our case, the occurrence of irAE during the first therapy was 5 days after the administration. Time to occurrence of the first irAE has been demonstrated to be associated with the recurrence of irAE. The average time for the first irAE was shorter in the recurrent patients compared to the non-recurrent patients (9 vs 15 weeks) (3). Surprisingly, in our case, no irAE happened during the ICI re-challenge therapy.

This case report has identified several limitations including incomplete estimation of PD-L1 expression and tumor mutation burden. Furthermore, individual study presents a potential for biased conclusions. Therefore, prospective studies are necessary to determine the efficacy and safety of ICI-based rechallenges in RCC patient post-recovery from adverse events, to gain a more comprehensive understanding of this therapeutic option.

In conclusion, re-challenging with immunotherapy after irAEs is an emerging strategy that offers the potential for additional clinical benefits to previously responding patients. However, careful patient selection and monitoring are essential to maximize the safety and efficacy of this approach. As cancer immunotherapy continues to evolve, refining the best approach to manage irAEs will be an essential component of patient care.

## Data availability statement

The original contributions presented in the study are included in the article/supplementary material. Further inquiries can be directed to the corresponding author.

## Ethics statement

Written informed consent was obtained from the individual(s) for the publication of any potentially identifiable images or data included in this article. Written informed consent was obtained from the participant/patient(s) for the publication of this case report.

## Author contributions

SH: Investigation, Writing – review & editing. LW: Writing – original draft. YS: Data curation, Writing – review & editing. HD: Data curation, Writing – review & editing. XY: Supervision, Writing – review & editing.
